# Using a Large Language Model (ChatGPT‐4o) to Assess the Risk of Bias in Randomized Controlled Trials of Medical Interventions: Interrater Agreement With Human Reviewers

**DOI:** 10.1002/cesm.70048

**Published:** 2025-09-10

**Authors:** Christopher James Rose, Julia Bidonde, Martin Ringsten, Julie Glanville, Thomas Potrebny, Chris Cooper, Ashley Elizabeth Muller, Hans Bugge Bergsund, Jose F. Meneses‐Echavez, Rigmor C. Berg

**Affiliations:** ^1^ Center for Epidemic Interventions Research Norwegian Institute of Public Health Oslo Norway; ^2^ Division of Health Services Norwegian Institute of Public Health Oslo Norway; ^3^ School of Rehabilitation Science University of Saskatchewan Saskatoon Saskatchewan Canada; ^4^ Cochrane Sweden Skåne University Hospital, Lund University Lund Sweden; ^5^ Glanville.info York UK; ^6^ Section Evidence‐Based Practice Western Norway University of Applied Sciences Bergen Norway; ^7^ Bristol Medical School University of Bristol Bristol UK; ^8^ Facultad de Cultura Física, Deporte y Recreación Universidad Santo Tomás Bogotá Colombia; ^9^ UiT The Arctic University of Tromsø Tromsø Norway

**Keywords:** artificial intelligence, ChatGPT, evidence synthesis, large language model, LLM, risk of bias, RoB

## Abstract

**Background:**

Risk of bias (RoB) assessment is a highly skilled task that is time‐consuming and subject to human error. RoB automation tools have previously used machine learning models built using relatively small task‐specific training sets. Large language models (LLMs; e.g., ChatGPT) are complex models built using non‐task‐specific Internet‐scale training sets. They demonstrate human‐like abilities and might be able to support tasks like RoB assessment.

**Methods:**

Following a published peer‐reviewed protocol, we randomly sampled 100 Cochrane reviews. New or updated reviews that evaluated medical interventions, included ≥ 1 eligible trial, and presented human consensus assessments using Cochrane RoB1 or RoB2 were eligible. We excluded reviews performed under emergency conditions (e.g., COVID‐19), and those on public health or welfare. We randomly sampled one trial from each review. Trials using individual‐ or cluster‐randomized designs were eligible. We extracted human consensus RoB assessments of the trials from the reviews, and methods texts from the trials. We used 25 review‐trial pairs to develop a ChatGPT prompt to assess RoB using trial methods text. We used the prompt and the remaining 75 review‐trial pairs to estimate human‐ChatGPT agreement for “Overall RoB” (primary outcome) and “RoB due to the randomization process”, and ChatGPT‐ChatGPT (intrarater) agreement for “Overall RoB”. We used ChatGPT‐4o (February 2025) throughout.

**Results:**

The 75 reviews were sampled from 35 Cochrane review groups, and all used RoB1. The 75 trials spanned five decades, and all but one were published in English. Human‐ChatGPT agreement for “Overall RoB” assessment was 50.7% (95% CI 39.3%–62.0%), substantially higher than expected by chance (*p* = 0.0015). Human‐ChatGPT agreement for “RoB due to the randomization process” was 78.7% (95% CI 69.4%–88.0%; *p* < 0.001). ChatGPT‐ChatGPT agreement was 74.7% (95% CI 64.8%–84.6%; *p* < 0.001).

**Conclusions:**

ChatGPT appears to have some ability to assess RoB and is unlikely to be guessing or “hallucinating”. The estimated agreement for “Overall RoB” is well above estimates of agreement reported for some human reviewers, but below the highest estimates. LLM‐based systems for assessing RoB may be able to help streamline and improve evidence synthesis production.

## 1 Introduction

Evidence syntheses, including systematic reviews and health technology assessments, often evaluate risk of bias (RoB) in randomized trials and nonrandomized studies. Trials that use inadequate randomization methods or allocation concealment [1, 2], for example, may overestimate treatment effects. Cochrane developed its own RoB tool in 2011 [3], which was later revised in 2019 as RoB2 [4].

Assessing RoB is resource‐intensive, requiring reviewers to interpret study methods accurately, understand trial design and reporting, proficiently apply RoB tools, and integrate assessments into evidence syntheses. Even experienced reviewers make errors and disagree with one another, potentially introducing bias. For example, previous studies have estimated human–human agreement of 16%–81% when conducting RoB assessments [5, 6].

The main potential benefits of machine learning and artificial intelligence (AI) tools to perform or support review tasks include reduced human effort and lower costs; reduced time to project completion [7]; more consistent performance compared to human reviewers; improved repeatability (i.e., being able to repeat an assessment at a later date to verify results); and being able to redirect human effort to tasks that cannot be performed by machines, such as those requiring high levels of creativity or empathy.

The role of automation in systematic review production has been explored for over a decade [8]. Semi‐automated tools like RobotReviewer [9] have been available to aid RoB assessment for some time, but are based on relatively simple models built using relatively small task‐specific training sets. We sought to investigate whether a large language model (LLM) such as ChatGPT (OpenAI, San Francisco, CA, USA), which is trained on an Internet‐scale non‐task‐specific training set, could be used to assess RoB of trials.

This article presents the results for a prespecified study on the agreement between human consensus‐based RoB assessments of trials of medical interventions and assessments made using ChatGPT. Further information is available in our protocol [10].

## 2 Methods

We performed the study according to the protocol; minor deviations are reported below. The unit of analysis was trial. We assessed agreement between human consensus‐based RoB assessments of trials obtained from Cochrane reviews and RoB assessments made by ChatGPT using trial methods text. We prespecified using methods text alone because our protocol was developed when ChatGPT's context window imposed a limit of approximately 3000 words, precluding the use of full trial reports and additional materials such as trial protocols, supplementary materials, and ancillary publications. Although ChatGPT was updated during the study to substantially enlarge its context window and allow files to be uploaded, we chose not to deviate from protocol to maintain methodological integrity and avoid potential research waste from managing comprehensive trial documentation if ChatGPT proved unable to assess risk of bias.

**Figure 1:**
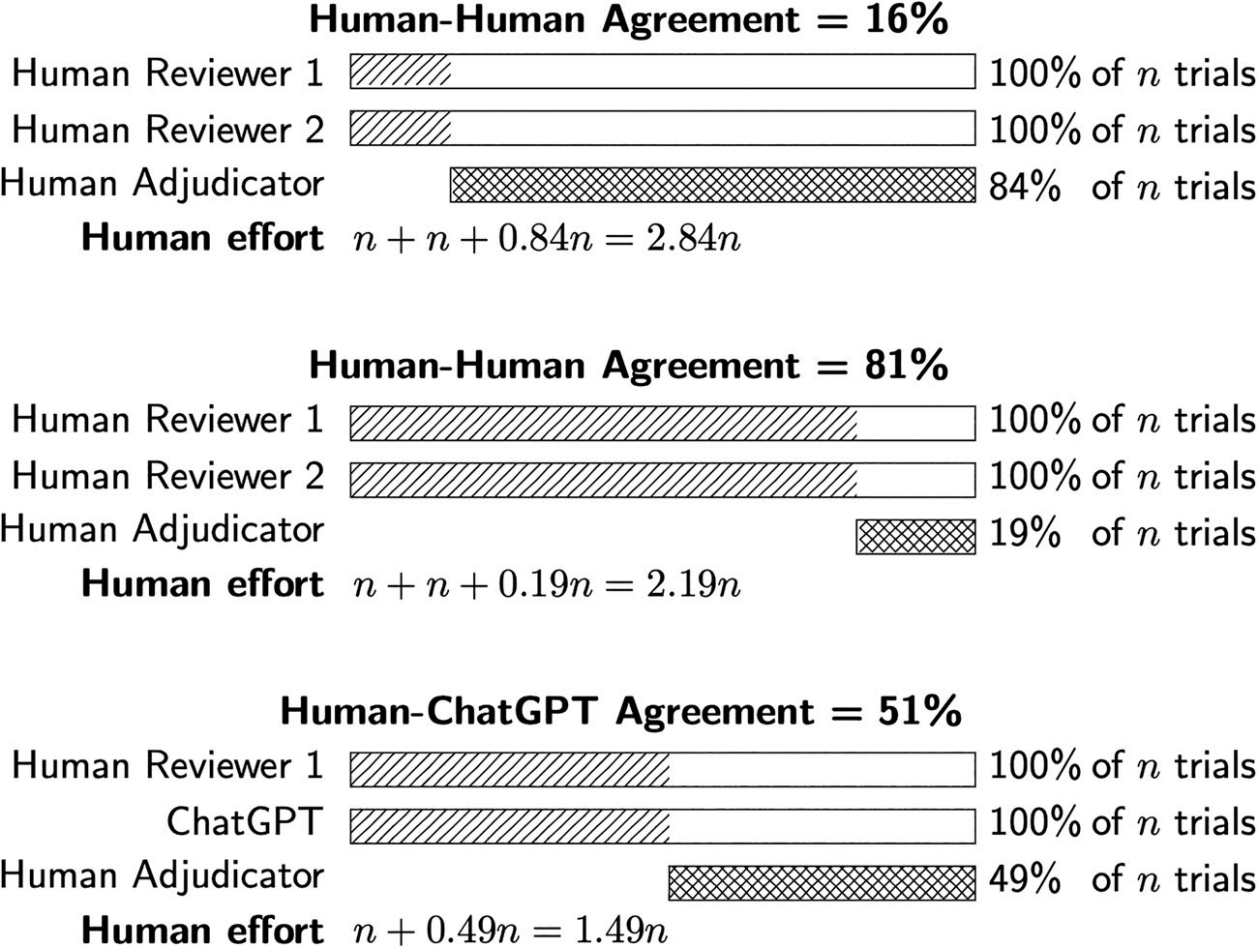
Human effort under human‐adjudicated RoB assessment. Under a simplified model of human‐adjudicated RoB assessment, two humans or a human and ChatGPT must each assess 100% of *n* trials for RoB, agreeing on some percentage of trials (indicated by the hatching). A human adjudicator must assess each trial for which agreement was not reached (crosshatching). Irrespective of whether human–human agreement is relatively low (top panel) or high (middle panel), meaningful reductions in human effort could be achieved using human‐adjudicated human–ChatGPT assessment (bottom panel).

We report the study following the Guidelines for Reporting Reliability and Agreement Studies (GRRAS [11]) checklist (see Supporting Information S1). We evaluated ChatGPT‐4o (February 2025) without using fine‐tuning, retrieval augmented generation, or changing any parameters such as temperature. We used that model to help make Figure 1, and we used ChatGPT and Claude Sonnet 4 (June 2025; Anthropic PBC, San Francisco, CA, USA) to help edit the article in response to peer reviews.

### 2.1 Eligibility Criteria

The eligibility criteria for reviews and trials are given in the protocol and summarized in Table 1.

**Table 1:** Summary of review and trial inclusion and exclusion criteria.

	Inclusion criteria	Exclusion criteria
Systematic reviews	Effectiveness of medical interventions Include ≥ 1 cluster or individually randomized parallel two‐arm RCT Human consensus‐based RoB assessments Use of RoB1 or an appropriate RoB2 tool^a^ Overall RoB assessment at the level of trial or primary outcome for benefit Published in any language	Use of heavily modified versions of the RoB tools Reviews performed under emergency conditions Public health or welfare reviews Reviews that used automation to assess RoB
Trials	Effectiveness of medical interventions Cluster‐ or individually randomized parallel two‐arm RCTs Primary outcome assesses benefit Published in any language^b^	Primary outcome assesses harm Estimates the effect of starting and adhering to treatment Inappropriate RoB tool used in review Not possible to identify a methods section

Abbreviations: RCT, randomized controlled trial; RoB, risk of bias.

^a^ Appropriate RoB2 tools include the RoB2 tool for individually‐randomized parallel‐group trials and the corresponding tool for cluster‐randomized trials.

b We planned to exclude trials published in a language we could not read if we were unable to determine the methods section of the trial.

### 2.2 Data Sources and Management

We performed a systematic literature search of the Cochrane Database of Systematic Reviews to identify potentially eligible Cochrane reviews of interventions published or updated between October 2011 (the date the RoB1 tool was published in the BMJ) and June 2023 (administrative cutoff). From the systematic reviews identified through our literature search, we randomly selected reviews using computer‐generated random numbers in batches of 10 (see Section Protocol and Section 2.10).

### 2.3 Selection

Reviews and trials were screened against the inclusion and exclusion criteria by two authors in duplicate in four stages: title and abstract (systematic reviews), full text (systematic reviews), title and abstract (trials), and finally full text (trials). Any disagreements were resolved by consensus or adjudication by a third author.

### 2.4 Outcomes

The protocol describes the outcomes and our rationale for specifying them. Briefly, the primary outcome was human–ChatGPT agreement in “Overall RoB” assessment, with ChatGPT RoB assessments made using trial methods text alone rather than the wider range of information that human reviewers may use to assess RoB (e.g., trial report, supplementary materials, trial protocol, statistical analysis plan, ancillary trial reports, conference abstracts). The protocol explains how we defined “Overall RoB” for RoB1 assessments.

There is evidence that humans overuse the “uncertain”/“some concerns” categories [12, 13], so we specified secondary outcomes to assess agreement between dichotomized assessments of RoB: low or unclear RoB versus high RoB, and low versus unclear or high RoB.

Research shows that trials tend to report exaggerated effect estimates if they are judged to have used inadequate randomization methods [1]. Randomization is key to being able to draw strong causal inferences about treatment effects. We therefore defined one exploratory domain‐level outcome, human‐ChatGPT agreement for “RoB due to the randomization process”, which is addressed by RoB1 and RoB2.

### 2.5 Data Extraction

We extracted data on the included reviews, trials, and the human consensus RoB assessments published in the Cochrane reviews using Google Sheets (Google Inc., Mountain View, CA, USA). We preferentially copied methods text from HTML versions of the trial reports or from PDFs. We did not correct any artefacts that arose from copying (e.g., words broken by hyphenation, reference numbers, non‐text characters).

### 2.6 Prompt Engineering

Between June 2024 and October 2024, we iteratively developed a prompt to request ChatGPT to assess “Overall RoB” and “RoB due to the randomization process” using trial methods text. We (CJR, HBB, JFME, JB) used 25 of the 100 included trials and their associated human RoB assessments to develop a prompt, drawing on guidance for best practice in prompt engineering [14]. We used a range of approaches, and in particular avoided developing “leading” prompts. For example, candidate prompts were developed by:

- transcribing the flow diagrams in the RoB2 guidance into a prompt;
- uploading that guidance and other RoB publications and asking ChatGPT to use them to assess RoB in trial methods text; and
- uploading that guidance and telling ChatGPT that it is an expert artificial intelligence researcher with experience in systematic reviewing, and that it should use the uploaded guidance to write a prompt for ChatGPT to assess trial methods text for RoB.

Several changes and apparent improvements to ChatGPT were made by OpenAI during the project, notably with respect to the underlying LLM, so we decided to model what many systematic reviewers might do, which is to use the most advanced model available at no cost. We chose the prompt that achieved the highest agreement between ChatGPT and human consensus RoB assessments. The prompt engineering work and all subsequent ChatGPT RoB assessments used the GPT‐4o model.

### 2.7 Power Calculation

We used simulation to determine that RoB assessments for 75 trials would provide at least 90% power at the 95% significance level (see Section Protocol).

### 2.8 ChatGPT‐Based RoB Assessment

To avoid biasing our results, we excluded the 25 trials used for prompt engineering from subsequent work on assessing human‐ChatGPT agreement (similar to using distinct “training” and “test” sets). We used ChatGPT to assess “Overall RoB” and “RoB arising from the randomization process” for each of the remaining 75 trials using the selected prompt (see Section 2.6); we did not use any additional prompts alongside the selected prompt. To facilitate the assessment of ChatGPT‐ChatGPT agreement, we obtained ChatGPT assessments in duplicate. This was done by a single author (CJR) rather than two different authors as planned, who ensured that a new chat session was used for each assessment to try to prevent ChatGPT from using information from previous assessments (which could bias its assessments and hence estimated agreement). We anticipated being able to get ChatGPT to respond with single‐word assessments, but this was not possible. We recorded ChatGPT's RoB assessments and responses in full. We did not blind those using ChatGPT to the human assessments of RoB (see Section Protocol).

### 2.9 Statistical Analyses

We summarized the results for the primary outcome as a confusion matrix (contingency table) and computed expected agreements under random assessment, observed agreements with two‐sided 95% confidence intervals (CIs), *p* values testing null hypotheses of random assessment, and Cohen's *κ* values with two‐sided 95% CIs (bootstrapped using 1000 replications) for all outcomes. If two raters assign three RoB categories randomly with equal probability, the expected agreement would be 33.3%; the expected agreements we present account for raters assigning RoB categories randomly with possibly unequal probabilities. We interpreted Cohen's *κ* per Landis and Koch [15] using words such as “fair” (for 0.21 ≤ *κ *≤ 0.40) and “moderate” (for 0.41 ≤ *κ *≤ 0.60). We used the *p* < 0.05 significance criterion throughout. We performed statistical analyses using Stata 18 (StataCorp LLC, College Station, Texas, USA). The statistician was not blinded.

We planned to perform subgroup analyses to compare agreement for reviews that used RoB1 versus RoB2, trials published before versus after the Consolidated Standards for Reporting of Trials (CONSORT [16]) were introduced in 1996, and trials published in English versus other languages, but these analyses were not possible (see Section [Link], Protocol).

### 2.10 Protocol Deviations

In addition to the minor deviations noted previously, we modified the batch sampling procedure. We sampled trials from review forest plots using a computer‐generated random number to index the selected trial. We had planned to discard an already‐screened review if the trial randomly selected from it did not meet the inclusion criteria or data could not be extracted as planned (e.g., some older trials could only be obtained as scanned images or hard copies from libraries). We revised the sampling strategy during screening and data extraction for the 25 review‐trial pairs used for prompt engineering to prevent wasting time discarding already‐screened reviews. Instead of discarding an already‐screened review when a trial was unusable, we moved to the adjacent trial in the forest plot by incrementing the random number by one (or decrementing by one if the selected trial was the last in the plot). We then screened the trial at that new position and attempted to extract data, repeating the process until a usable trial was found or the review had to be discarded as containing no usable trials. For example, if the fourth trial in a forest plot had been randomly selected but data extraction was not possible, we would screen and extract data from the 5th trial. The revised procedure should only bias our sample with respect to the distribution of RoB if trials were sorted by RoB, which they were not.

We planned to obtain two ChatGPT assessments for each trial and estimate human‐ChatGPT agreement using one randomly‐chosen ChatGPT assessment for each trial and estimate ChatGPT‐ChatGPT agreement using both ChatGPT assessments. In practice, we obtained one ChatGPT assessment for each trial and used these to estimate human‐ChatGPT agreement, and then obtained a second set of ChatGPT assessments to estimate ChatGPT‐ChatGPT agreement.

## 3 Results

### 3.1 Prompt

The best‐performing prompt (Box 1) agreed with 10 (40%; 95% CI 21%–61%) of the 25 human assessments of “Overall RoB”, and 14 (56%; 95% CI 35%–76%) of the human assessments of “RoB due to the randomization process”. We had planned to ask a single follow‐up question if needed, but this was never necessary.

Box 1 **The selected ChatGPT prompt used to assess risk of bias.**

You are a professional systematic review researcher with expertise in assessing risk of bias using the Cochrane Risk of Bias tool. I will provide you with a methods section from a randomized controlled trial (RCT) of a medical intervention, delimited by triple quotes. You will use the Cochrane Risk of Bias tool to assess the risk of bias, based on that methods section. Limit your answers to “high”, “low”, “unclear”, or “insufficient information”.

For each domain of the Cochrane Risk of Bias tool, provide your assessment. The domains to be assessed are:

1. Random sequence generation (selection bias)
2. Allocation concealment (selection bias)
3. Blinding of participants and personnel (performance bias)
4. Blinding of outcome assessment (detection bias)
5. Incomplete outcome data (attrition bias)
6. Selective reporting (reporting bias)
7. Other bias (consider other potential sources of bias)

If there is insufficient information in the methods section to assess risk of bias for a given domain, write “insufficient information” for that domain.

Finally, you will assess the overall risk of bias. The rules for assessing the overall risk of bias are as follows:

- The RCT is at low overall risk of bias if it is judged to be at low risk of bias for all domains.
- The RCT is at unclear overall risk of bias if it is judged to be at unclear risk of bias for at least one domain, and it is not judged to be at high risk of bias for any of the domains.
- Otherwise, the RCT is at high overall risk of bias.

# Methods section

"""< insert text here >"""

### 3.2 Reviews and Trials

Table 2 summarizes the characteristics of the 75 review‐trial pairs used to assess agreement. The reviews were all published in English, as were all but one of the trials (Spanish). All reviews employed RoB1 (RoB2 was only released towards the end of our sampling timeframe and appears to have experienced slow initial adoption). The included trials were published between April 1977 and March 2021 (median: June 2008; interquartile range [IQR]: June 2004 to May 2012). Ninety‐five percent of the trials were published after the CONSORT statement was published.

**Table 2:** Characteristics of the included reviews and trials.

Reviews		
Included		75
Publication or update month/year	Median (IQR)	05/2017 (12/2014 to 07/2019)
Cochrane review groups	Anesthesia	6 (8.0%)
(by frequency)	Gut	6 (8.0%)
	Vascular	5 (6.7%)
	Neonatal	4 (5.3%)
	Pregnancy and Childbirth	4 (5.3%)
	Other (30 review groups)	50
Risk of bias tool	RoB1	75 (100%)
	RoB2	0 (0%)
Trials		
Included		75
Publication month/year	Median (IQR)	06/2008 (06/2004 to 05/2012)
Published after CONSORT		71 (95.0%)
Published in English		74 (98.7%)

### 3.3 Human‐ChatGPT Agreement for “Overall RoB”

Table 3 presents the confusion matrix for “Overall RoB”. Trials that humans assessed to be at low RoB were assessed by ChatGPT to be at low or uncertain RoB with similar frequencies. Trials that humans assessed to be at uncertain RoB were assessed by ChatGPT to be at low, uncertain, or high RoB, again with similar frequencies, although ChatGPT tended to assess these trials as being of low RoB. Trials that humans assessed to be at high RoB were assessed by ChatGPT to be at low, uncertain, or high RoB, with just over a third of assessments agreeing with humans.

**Table 3:** Confusion matrix for “Overall RoB” assessment.

		ChatGPT		
		Low	Uncertain	High
Human	Low	7/75 (9.3%)	5/75 (6.7%)	0/75 (0.0%)
	Uncertain	5/75 (6.7%)	3/75 (4.0%)	2/75 (2.7%)
	High	12/75 (16.0%)	13/75 (17.3%)	28/75 (37.3%)

Table 4 presents expected and observed agreements and estimates of Cohen's *κ*. If humans and ChatGPT were assessing RoB at random, then we would expect to observe an agreement of 37.1% (Table 4). This possibility is excluded at the prespecified significance level by the observed agreement of 50.7% (95% CI 39.3%–62.0%; *p* = 0.0015). Cohen's *κ* was estimated to be 0.215 (95% CI 0.072–0.359), or “slight” (95% CI “poor” to “fair”) agreement.

**Table 4:** Agreement in risk of bias assessments.

		Agreement (%)			
	N	Expected	Observed [95% CI]	*p* value	Cohen's *κ* [95% CI]
Primary outcome					
Human–ChatGPT agreement	75	37.1	50.7 [39.3–62.0]	0.0015	0.215 [0.072–0.359]
Subgroup analyses					
Reviews published					
Before CONSORT	4	50.0	50.0 [NC]	NC	0.00
After CONSORT	71	36.6	50.7 [39.2–62.2]	0.0016	0.222 [0.078–0.365]
Reviews that used					
RoB1	75	37.1	50.7 [39.3–62.0]	0.0015	0.215 [0.072–0.359]
RoB2	0	NC	NC	NC	NC
Trials published in					
English	74	36.6	50.0 [38.6–61.4]	0.0019	0.211 [0.073–0.349]
Another language	1	NC	NC	NC	NC
Secondary outcomes					
Low and unclear combined	75	45.9	64.0 [53.0–75.0]	0.0002	0.335 [0.168–0.502]
Unclear and high combined	75	62.2	70.7 [60.7–80.6]	0.0164	0.223 [0.006–0.441]
ChatGPT‐ChatGPT agreement	75	33.2	74.7 [64.8–84.6]	< 0.0001	0.621 [0.476–0.765]
Exploratory outcome					
Human–ChatGPT agreement for RoB due to randomization	75	53.9	78.7 [69.4–88.0]	< 0.0001	0.537 [0.336–0.738]

*Note:* Expected agreement is the percentage of risk of bias assessments that would be expected to agree if both raters (e.g., human reviewers and ChatGPT) assessed risk of bias at random. The *p* value tests the hypothesis of no difference between expected and observed agreement.

Abbreviation: NC, not computable.

Only one subgroup for each of the planned comparisons contained enough RoB assessments to support analysis, so subgroup comparisons could not be made (Table 4).

### 3.4 Low or Unclear Versus High RoB, and Low Versus Unclear or High RoB, for “Overall RoB”

Human‐ChatGPT agreement was estimated to be higher if the RoB categories low and unclear, or unclear and high, are merged (see Section 2.4 and Table 4). Agreement was estimated to be 70.7% (95% CI 60.7%–80.6%; *p* = 0.0164) if the “unclear” and “high” categories are merged, similar to the highest estimates of human–human agreement.

### 3.5 Human–ChatGPT Agreement for “RoB due to the Randomization Process”

Human–ChatGPT agreement was substantially higher than for “Overall RoB” (78.7%; 95% CI 69.4%–88.0%; *p* < 0.0001). This likely reflects that randomization methods are more objectively assessable than the other domains, which often require subjective judgment about adequacy of reporting or implementation.

### 3.6 ChatGPT–ChatGPT Agreement for “Overall RoB”

Agreement was estimated to be 74.7% (95% CI 64.8%–84.6%; *p* < 0.0001), which is similar to, and perhaps better than, the highest estimates of human–human agreement.

## 4 Discussion

### 4.1 Main Findings

ChatGPT‐4o has some ability to assess RoB in randomized trials. Its assessments of “Overall RoB”, made in a few seconds from trial methods texts, agreed with human consensus assessments in around half of the 75 trials. Across the outcomes studied, agreement is higher than or similar to the 16%–81% agreement previously reported between human reviewers and is unlikely to be due to chance (e.g., “hallucination”). That nontrivial human‐ChatGPT agreement can be achieved from ChatGPT assessments of methods text may be useful: it is relatively simple for a human or automated system to identify and extract the methods section of a trial report, so useful LLM‐based tools could plausibly be built around the approach.

Figure 1 illustrates the human effort required according to a simplified model of human‐adjudicated RoB assessment. In the top panel, two human reviewers each assess all trials for RoB and agree on 16%; a human adjudicator assesses 84% of trials for which the human reviewers disagree. With *n* trials, human effort is required for 2.84*n* trial assessments. In the middle panel, human‐human agreement is 81%, and human effort is required for 2.19*n* trial assessments. In the bottom panel, ChatGPT replaces a human reviewer. If human‐ChatGPT agreement is 51%, human adjudicator effort is required to assess 49% of the trials, and, in total, human effort is required for 1.49*n* trial assessments. This is 68% of the effort required for human‐adjudicated assessment by human reviewers who agree for 81% of trials, and 52% of the effort required for human reviewers who agree for 16% of trials. This suggests a meaningful reduction in human effort could be achieved even if human–ChatGPT agreement is modest compared to human–human agreement. A key insight is that, under this simplified model, human‐adjudicated human–human RoB assessment cannot use less human effort than human‐adjudicated human–ChatGPT RoB assessment, even if human‐human agreement is 100% and human–ChatGPT agreement is 0%.

### 4.2 Our Findings in the Context of Previous Research

LLM assessment of RoB has been studied previously [17, 18, 19, 20], although little previous research was prespecified. Our findings are broadly consistent with a smaller (*N* = 30) study by Lai et al. [19], which published estimates of agreement all somewhat higher than ours. Unlike our study, the human RoB assessments were obtained by the investigators rather than independent reviewers. These and our findings appear to differ markedly from those of a much larger (*N* = 5993 and *N* = 28,150) highly automated study of several LLMs by Šuster et al. [20], which found that LLMs generally fail to exceed trivial baselines. However, there are multiple important methodological differences between our study and those of Lai et al. and Šuster et al., which preclude exact comparison.

Interestingly, Šuster et al. used prompts that included signaling questions, similar to the more complex prompts we evaluated, but found as we did that they appear to perform substantially worse than the simple prompt we selected. One possibility is that Šuster et al. chose a suboptimal prompt, even though they appear to have followed best practices for prompt engineering. Another possibility is that any discrepancy in findings is due to their use of a complex prompt with the gpt‐3.5‐turbo LLM, and our use of a simple prompt with the GPT‐4o LLM (in general, we found simpler prompts to agree better with human consensus assessments).

### 4.3 Strengths and Limitations

This study was prespecified performed according to a published and peer‐reviewed protocol; we report and justify minor protocol deviations. We estimated agreement using human consensus assessments from Cochrane reviews—among the most thorough evidence syntheses in healthcare—using risk of bias assessments conducted by reviewers who were independent of the present study [21, 22]. We included trials spanning five decades, suggesting our findings are applicable to a broad range of trials that are likely to be included in reviews of medical treatments. We used outcomes and analyses that are useful and address anticipated risks and limitations (see Section Protocol).

The main limitations of the study are that we only studied agreement for “Overall RoB” and “RoB due to the randomization process” (i.e., did not study other domains), and ChatGPT's assessments were made using methods text alone (rather than full trial reports and possibly additional material, which human reviewers would usually have access to). The protocol describes our rationale for these choices in more detail. Domain‐level assessments are beyond the scope of the present work, but we may study them in the future. While our analysis was limited to trials from Cochrane reviews, the trials themselves represent studies that could be included in any systematic review, suggesting our findings have some generalizability beyond the Cochrane context. We considered at the protocol stage the possibility that ChatGPT could have been trained on the Cochrane reviews we would include in our sample and could demonstrate agreement by recalling memorized assessments rather than performing the assessment task. This is another possible explanation for ChatGPT's relatively strong performance on the randomization domain assessments (which were reported clearly in the Cochrane reviews) and the relatively low agreement on overall RoB assessments (which is not one of the RoB1 domains but might be reported clearly in some cases).

### 4.4 Implications for Practice and Research

Should reviewers use ChatGPT to assess RoB in the future? RoB assessment is a nontrivial task that is important and subjective (highly trained reviewers can disagree). We found that ChatGPT has some ability to perform this task and could, in principle, be used to save time and resources in evidence synthesis production. However, the decision to adopt ChatGPT or any other tool depends on a careful evaluation of the risks and benefits for a particular setting.

Our data (see Data availability statement) may be useful for future research, though we refer readers to the much larger data set published by Šuster et al. [20], but note that it is unlikely to have been thoroughly checked by humans.

Thomas and others have envisioned how reviewing could be automated [23, 24, 25]. We hope our work illustrates how researchers might assess potential future AI developments, such as agentic AI, in which AIs interact with systems such as databases and authoring platforms to achieve larger goals, such as drafting an entire review. Rather than copy and paste methods text as we did, agentic AIs could perform RoB assessment as part of a largely automated systematic reviewing process using trial data identified with minimal human guidance; we envisage that this would include all documentation related to a trial, not just the methods text. RoB assessment could be performed by several different independent AIs, with disagreements flagged for human attention.

Although existing tools like RobotReviewer facilitate automated RoB assessments within specific frameworks such as RoB1, LLMs' broader language capabilities may complement or replace such tools by potentially supporting newer assessment frameworks such as RoB2, warranting continued evaluation of both traditional and LLM‐based approaches.

## 5 Conclusions

ChatGPT‐4o appears to have some ability to assess RoB and is unlikely to be guessing or “hallucinating”: *p* values and 95% confidence intervals on observed agreements all exclude chance agreement. The estimated agreement for “Overall RoB” is well above the agreement reported in the literature between some human reviewers, but clearly below the approximately 80% reported at the higher end. Agreement for “RoB due to the randomization process” is substantially better and comparable with human–human agreement. LLM‐based systems for assessing RoB may be able to help streamline and improve evidence synthesis production.

## Protocol

Rose, C. J., Bidonde, J., Ringsten, M. et al., “Using a Large Language Model (ChatGPT) to Assess Risk of Bias in Randomized Controlled Trials of Medical Interventions: Protocol for a Pilot Study of Interrater Agreement With Human Reviewers, BMC Med. Res. Methodol. 25, 182 (2025), https://doi.org/10.1186/s12874-025-02631-0.

Rose, C. J., Ringsten, M., Bidonde J., et al., “Using a Large Language Model (ChatGPT) to Assess Risk of Bias in Randomized Controlled Trials of Medical Interventions: Protocol for a Pilot Study of Interrater Agreement With Human Reviewers,” (September 8, 2023), Available at Research Square https://doi.org/10.21203/rs.3.rs-3288515/v1.

## Author Contributions

**Christopher James Rose:** conceptualization, methodology, data curation, investigation, validation, formal analysis, supervision, project administration, writing – original draft, writing – review and editing. **Julia Bidonde:** methodology, data curation, investigation, validation, writing – review and editing. **Martin Ringsten:** data curation, investigation, validation, writing – review and editing. **Julie Glanville:** data curation, investigation, validation, writing – review and editing. **Thomas Potrebny:** investigation, writing – review and editing. **Chris Cooper:** methodology, data curation, investigation, validation, writing – review and editing. **Ashley Elizabeth Muller:** conceptualization, methodology, data curation, investigation, supervision, project administration, writing – original draft, writing – review and editing. **Hans Bugge Bergsund:** methodology, data curation, investigation, writing – review and editing. **Rigmor C. Berg:** data curation, methodology, investigation, supervision, project administration, writing – review and editing.

## Conflicts of Interest

The authors declare no conflicts of interest.

## Reporting Guideline

We followed the guidelines for reporting reliability and agreement studies (GRRAS; see Supporting Information S1).

## 1 Peer Review

The peer review history for this article is available at https://www.webofscience.com/api/gateway/wos/peer-review/10.1002/cesm.70048.

## Supporting information

GRRAS Checklist.

## Data Availability

Data and analysis code are available at https://doi.org/10.5281/zenodo.16926876. We are not able to publish the methods text extracted from trial reports due to copyright.

## References

[1] M. J. Page , J. P. T. Higgins , G. Clayton , J. A. C. Sterne , A. Hróbjartsson , and J. Savović , “Empirical Evidence of Study Design Biases in Randomized Trials: Systematic Review of Meta‐Epidemiological Studies,” PLoS One 11, no. 7 (2016): e0159267. 27398997 10.1371/journal.pone.0159267 PMC4939945

[2] K. F. Schulz , “Empirical Evidence of Bias: Dimensions of Methodological Quality Associated With Estimates of Treatment Effects in Controlled Trials,” Journal of the American Medical Association (Chicago, IL) 273, no. 5 (1995): 408–412. 10.1001/jama.273.5.408 7823387

[3] J. P. T. Higgins , D. G. Altman , P. C. Gotzsche , et al., “The Cochrane Collaboration's Tool for Assessing Risk of Bias in Randomised Trials,” BMJ 343 (October 2011): d5928. 22008217 10.1136/bmj.d5928 PMC3196245

[4] J. A. C. Sterne , J. Savović , M. J. Page , et al., “RoB 2: a Revised Tool for Assessing Risk of Bias in Randomised Trials,” BMJ 366 (August 2019): l4898. 31462531 10.1136/bmj.l4898

[5] N. Könsgen , O. Barcot , S. Heß , et al., “Inter‐Review Agreement of Risk‐of‐Bias Judgments Varied in Cochrane Reviews,” Journal of Clinical Epidemiology 120 (April 2020): 25–32. 31866473 10.1016/j.jclinepi.2019.12.016

[6] S. Minozzi , M. Cinquini , S. Gianola , M. Gonzalez‐Lorenzo , and R. Banzi , “The Revised Cochrane Risk of Bias Tool for Randomized Trials (RoB 2) Showed Low Interrater Reliability and Challenges in Its Application,” Journal of Clinical Epidemiology 126 (October 2020): 37–44. 32562833 10.1016/j.jclinepi.2020.06.015

[7] A. E. Muller , R. C. Berg , J. F. Meneses‐Echavez , et al., “The Effect of Machine Learning Tools for Evidence Synthesis on Resource Use and Time‐to‐Completion: Protocol for a Retrospective Pilot Study,” Systematic Reviews 12, no. 1 (January 2023): 7. 36650579 10.1186/s13643-023-02171-y PMC9843684

[8] G. Tsafnat , P. Glasziou , M. K. Choong , A. Dunn , F. Galgani , and E. Coiera , “Systematic Review Automation Technologies,” Systematic Reviews 3, no. 1 (December 2014): 74. 25005128 10.1186/2046-4053-3-74 PMC4100748

[9] I. J. Marshall , J. Kuiper , and B. C. Wallace , “Robotreviewer: Evaluation of a System for Automatically Assessing Bias in Clinical Trials,” Journal of the American Medical Informatics Association 23, no. 1 (January 2016): 193–201. 26104742 10.1093/jamia/ocv044 PMC4713900

[10] C. J. Rose , J. Bidonde , M. Ringsten , et al., “Using a Large Language Model (ChatGPT) to Assess Risk of Bias in Randomized Controlled Trials of Medical Interventions: Protocol for a Pilot Study of Interrater Agreement With Human Reviewers,” BMC Medical Research Methodology 25, no. 1 (July 2025): 182. 40745627 10.1186/s12874-025-02631-0 PMC12315198

[11] J. Kottner , L. Audigé , S. Brorson , et al., “Guidelines for Reporting Reliability and Agreement Studies (GRRAS) Were Proposed,” Journal of Clinical Epidemiology 64, no. 1 (January 2011): 96–106. 21130355 10.1016/j.jclinepi.2010.03.002

[12] L. Jørgensen , A. S. Paludan‐Müller , D. R. T. Laursen , et al., “Evaluation of the Cochrane Tool for Assessing Risk of Bias in Randomized Clinical Trials: Overview of Published Comments and Analysis of User Practice in Cochrane and Non‐Cochrane Reviews,” Systematic Reviews 5 (May 2016): 80. 27160280 10.1186/s13643-016-0259-8 PMC4862216

[13] A. Dechartres , L. Trinquart , I. Atal , et al., “Evolution of Poor Reporting and Inadequate Methods Over Time in 20 920 Randomised Controlled Trials Included in Cochrane Reviews: Research on Research Study,” BMJ 357 (June 2017): j2490. 28596181 10.1136/bmj.j2490

[14] OpenAI, “Best Practices for Prompt Engineering With OpenAI API,” OpenAI, accessed May 8, 2023, https://help.openai.com/en/articles/6654000-best-practices-for-prompt-engineering-with-openai-api.

[15] J. R. Landis and G. G. Koch , “The Measurement of Observer Agreement for Categorical Data,” Biometrics 33, no. 1 (March 1977): 159–174. 843571

[16] C. Begg , “Improving the Quality of Reporting of Randomized Controlled Trials. The CONSORT Statement,” Journal of the American Medical Association (Chicago, IL) 276, no. 8 (August 1996): 637–639. 10.1001/jama.276.8.637 8773637

[17] J. Barsby , S. Hume , H. A. Lemmey , J. Cutteridge , R. Lee , and K. D. Bera , “Pilot Study on Large Language Models for Risk‐of‐Bias Assessments in Systematic Reviews: A(I) New Type of Bias?,” BMJ Evidence‐Based Medicine 30, no. 1 (February 2025): 71–74. 10.1136/bmjebm-2024-112990 38782561

[18] A. Eisele‐Metzger , J. L. Lieberum , M. Toews , et al., “Exploring the Potential of Claude 2 for Risk of Bias Assessment: Using a Large Language Model to Assess Randomized Controlled Trials With RoB 2,” Research Synthesis Methods 16, no. 3 (July 2025): 491. 10.1017/rsm.2025.12 PMC12527486 41626932

[19] H. Lai , L. Ge , M. Sun , et al., “Assessing the Risk of Bias in Randomized Clinical Trials With Large Language Models,” JAMA Network Open 7, no. 5 (May 2024): e2412687. 38776081 10.1001/jamanetworkopen.2024.12687 PMC11112444

[20] S. Šuster , T. Baldwin , and K. Verspoor , “Zero‐ and Few‐Shot Prompting of Generative Large Language Models Provides Weak Assessment of Risk of Bias in Clinical Trials,” Research Synthesis Methods 15, no. 6 (November 2024): 988–1000. 39176994 10.1002/jrsm.1749

[21] M. Pollock , R. M. Fernandes , and L. Hartling , “Evaluation of AMSTAR to Assess the Methodological Quality of Systematic Reviews in Overviews of Reviews of Healthcare Interventions,” BMC Medical Research Methodology 17, no. 1 (March 2017): 48. 28335734 10.1186/s12874-017-0325-5 PMC5364717

[22] S. Dosenovic , A. Jelicic Kadic , K. Vucic , N. Markovina , D. Pieper , and L. Puljak , “Comparison of Methodological Quality Rating of Systematic Reviews on Neuropathic Pain Using Amstar and R‐AMSTAR,” BMC Medical Research Methodology 18, no. 1 (December 2018): 37. 29739339 10.1186/s12874-018-0493-y PMC5941595

[23] I. J. Marshall and B. C. Wallace , “Toward Systematic Review Automation: A Practical Guide to Using Machine Learning Tools in Research Synthesis,” Systematic Reviews 8, no. 1 (July 2019): 163. 31296265 10.1186/s13643-019-1074-9 PMC6621996

[24] J. Thomas , A. Noel‐Storr , I. Marshall , et al., “Living Systematic Reviews: 2. Combining Human and Machine Effort,” Journal of Clinical Epidemiology 91 (November 2017): 31–37. 28912003 10.1016/j.jclinepi.2017.08.011

[25] A. M. O'Connor , J. Clark , J. Thomas , et al., “Large Language Models, Updates, and Evaluation of Automation Tools for Systematic Reviews: A Summary of Significant Discussions At the Eighth Meeting of the International Collaboration for the Automation of Systematic Reviews (ICASR),” Systematic Reviews 13, no. 1 (November 2024): 290. 39605097 10.1186/s13643-024-02666-2 PMC11600926

